# Unlocking the potential of TIPS placement as a bridge to elective and emergency surgery in cirrhotic patients: a meta-analysis and future directions for endovascular resuscitation in acute care surgery

**DOI:** 10.1186/s13017-023-00498-4

**Published:** 2023-04-17

**Authors:** Ramiro Manzano-Nunez, Alba Jimenez-Masip, Julian Chica-Yanten, Abdelaziz Ibn-Abdelouahab, Massimo Sartelli, Nicola de’Angelis, Ernest E. Moore, Alberto F. García

**Affiliations:** 1 Universitat Autònoma de Barcelona, Barcelona, Spain; 2 Vall d’Hebron University Hospital, Barcelona, Spain; 3 Department of Surgery, Universidad Javeriana, Bogotá, Colombia; 4 Mohammed V University, Rabat, Morocco; 5 Department of Surgery, Macerata Hospital, Macerata, Italy; 6 Colorectal and Digestive Surgery Unit, Beaujon Hospital, Paris, Île-de-France, France; 7 Ernest E. Moore Shock Trauma Center and University of Colorado, Denver, CO USA; 8 Department of Surgery, Fundacion Valle del Lili, Cali, Colombia; 9 Clinical Research Center, Fundacion Valle del Lili, Cali, Colombia

**Keywords:** Liver cirrhosis, Portal hypertension, Transjugular intrahepatic portosystemic shunt, General surgery, Abdominal surgery, Outcomes

## Abstract

**Background:**

In this systematic review and meta-analysis, we examined the evidence on transjugular intrahepatic portosystemic shunt (TIPS) as a bridge to elective and emergency surgery in cirrhotic patients. We aimed to assess the perioperative characteristics, management approaches, and outcomes of this intervention, which is used to achieve portal decompression and enable the safe performance of elective and emergent surgery.

**Methods:**

MEDLINE and Scopus were searched for studies reporting the outcomes of cirrhotic patients undergoing elective and emergency surgery with preoperative TIPS. The risk of bias was evaluated using the methodological index for non-randomized studies of interventions, and the JBI critical appraisal tool for case reports. The outcomes of interest were: 1. Surgery after TIPS; 2. Mortality; 3. Perioperative transfusions; and 4. Postoperative liver-related events. A DerSimonian and Laird (random-effects) model was used to perform the meta-analyses in which the overall (combined) effect estimate was presented in the form of an odds ratio (summary statistic).

**Results:**

Of 426 patients (from 27 articles), 256 (60.1%) underwent preoperative TIPS. Random effects MA showed significantly lower odds of postoperative ascites with preoperative TIPS (OR = 0.40, 95% CI 0.22–0.72; I2 = 0%). There were no significant differences in 90-day mortality (3 studies: OR = 0.76, 95% CI 0.33–1.77; I2 = 18.2%), perioperative transfusion requirement (3 studies: OR = 0.89, 95% CI 0.28–2,84; I2 = 70.1%), postoperative hepatic encephalopathy (2 studies: OR = 0.97, 95% CI 0.35–2.69; I2 = 0%), and postoperative ACLF (3 studies: OR = 1.02, 95% CI 0.15–6.8, I2 = 78.9%).

**Conclusions:**

Preoperative TIPS appears safe in cirrhotic patients who undergo elective and emergency surgery and may have a potential role in postoperative ascites control. Future randomized clinical trials should test these preliminary results.

**Supplementary Information:**

The online version contains supplementary material available at 10.1186/s13017-023-00498-4.

## Background

Portal hypertension (PH) is a key driver of hepatic decompensation and mortality among patients with advanced chronic liver disease or cirrhosis. PH in cirrhotic patients occurs due to increased intrahepatic resistance that induces systemic hemodynamic disturbances [1]. When the hepatic venous pressure gradient (HVPG) rises up to 10 mmHg (i.e., clinically significant portal hypertension) [2], patients may develop gastroesophageal varices or other portosystemic collaterals, but even more importantly, hepatic complications such as ascites, hepatic encephalopathy, portal hypertensive bleeding or portal vein thrombosis [3].

Surgery, particularly major surgery, is one of the precipitants of acute decompensation in patients with cirrhosis and PH, and postoperative morbidity and mortality correlate with liver disease severity [4]. Consequently, surgery may be contraindicated in some patients with cirrhosis and PH. Therefore, surgeons often encounter challenging situations when evaluating patients with advanced cirrhosis who require elective or emergency surgeries in the context of acute care and general surgery. This is because the presence of advanced liver disease, such as portal hypertension, can impact the decision to perform a surgical procedure that would otherwise be performed without concern for complications and poor outcomes.

Transjugular intrahepatic portosystemic shunt (TIPS) has been used to manage complications related to PH, including portal hypertensive bleeding, ascites, and portal vein thrombosis [5]. It has been proposed that preoperative TIPS placement, by lowering portal pressure, would improve postoperative outcomes [5, 6]. Available evidence suggests that preoperative TIPS placement may be safe and could potentially reduce postoperative liver outcomes [7]. Unfortunately, we need more research to test the effects of TIPS in the field of surgery. Such research would be of particular interest to surgeons and other healthcare professionals caring for cirrhotic patients undergoing surgical procedures [8] and could inform clinical practice and guide future research efforts in this field.

To date, the role of TIPS in the preoperative setting of patients requiring elective and emergency surgery remains unclear. Moreover, little quantitative evidence is available to know the number of patients who can undergo surgery after preoperative TIPS and their associated postoperative outcomes; for example, postoperative liver-related events (LRE).

In this systematic review and meta-analysis, we examined the evidence on using transjugular intrahepatic portosystemic shunt (TIPS) placement as a bridge to elective and emergency surgery in cirrhotic patients. We aimed to assess the perioperative characteristics, management approaches, and outcomes of this intervention, which is used to achieve portal decompression and enable the safe performance of surgery. We hypothesized that preoperative TIPS deployment reduces morbidity and mortality in cirrhotic patients undergoing surgery.

## Methods

The present meta-analytic review adhered to the principles from the Cochrane Handbook of Systematic Reviews of Interventions and was reported according to the PRISMA [9] and “Meta-analysis of Observational Studies in Epidemiology” (MOOSE) reporting guidelines [10].

To achieve our main objective, this SR and MA answered the following questions:

1. What is the available evidence on the use of TIPS as a bridge to elective and emergency surgery in cirrhotic patients?
2. What are the reported effects of preoperative TIPS placement on the outcomes of cirrhotic patients undergoing elective and emergency surgery?

Although not registered in PROSPERO, a protocol prepared before the review kickoff was used to guide the execution of the systematic review.

### Inclusion criteria

Studies were considered eligible for inclusion if they reported the characteristics and outcomes of cirrhotic patients undergoing elective and emergency surgery with preoperative TIPS.

### Exclusion criteria

Studies on cirrhotic patients undergoing hepatic surgery (i.e., hepatic resection or liver transplantation) and those involving subjects with non-cirrhotic portal hypertension were excluded.

### Types of studies

We included observational studies of any type (case series, cohort studies, case–control studies). Case reports were also considered eligible for inclusion in this SR. Narrative reviews, commentaries, and editorials without patient data were not considered eligible for inclusion.

### Types of patients and interventions

The participants were patients with liver cirrhosis of any etiology requiring elective or emergency surgery (i.e., extrahepatic cancer requiring resection, hernia surgery, cholecystectomy, among others) and undergoing preoperative TIPS to achieve portal decompression as means of mitigating the risks associated with PH. Preoperative TIPS to achieve portal decompression was defined as TIPS created in anticipation to surgery either as a prophylactic strategy or to treat a liver-related event (LRE) in progress at the time when surgery was planned/scheduled.

### Outcomes

The outcomes of interest in this systematic review were: 1. Surgery following preoperative TIPS (# of patients who underwent surgery as planned following the preoperative TIPS procedure), 2. Mortality, 3. Perioperative transfusions requirements, and 4. Postoperative liver-related events (LRE). We intended to collect data on the following postoperative LREs (if available from primary studies): ascites, hepatic encephalopathy, portal hypertensive bleeding and acute on chronic liver failure (ACLF). We also registered the timeframe from TIPS to surgery and if the surgical procedure was elective or emergent.

### Search methods

An electronic database search strategy of the available literature was performed following experts’ recommendations. In addition, the literature search was planned according to the iterative process recommended by librarians from the National University of Singapore [11].

The literature search was performed in MEDLINE and Scopus from inception to 28 August 2022. The search included keywords related to the population/patients of interest (cirrhotic patients requiring surgical procedures) and the intervention of interest (TIPS). The electronic database searching was complemented by a snowball scanning of the references cited in the included studies. Complete electronic search strategies are available in the Additional file 1.

### Study selection

Results from the electronic search strategies were imported into Ryyan [12]. Then, titles and abstracts were initially screened by two authors (RM and JC) blindly and independently. In the title and abstract screening phase, potential articles were selected based on the inclusion and exclusion criteria previously defined. Articles that appeared relevant during the initial screening phase of the study were retrieved as full texts and subsequently reviewed by two authors (RM and JC), who blindly and independently applied inclusion and exclusion criteria to full texts for final eligibility and inclusion.

When two articles appeared to be reporting data from overlapping populations (i.e., different papers reporting data from the same population or the exact center/hospital during overlapping periods), the publication with the larger sample size or greater/deepest data granularity was selected for inclusion.

### Data collection

The full texts finally selected were reviewed in detail to collect data relevant to the topic of this SR. Data were extracted as reported in the selected studies and imported into a pre-designed data collection form, in which the following data were registered: authors, year of publication, region/hospital of origin, study type, recruitment period, number of patients, relevant demographic and clinical data, TIPS procedure characteristics, conditions requiring surgery, type of surgery performed (elective or emergent), cirrhosis etiology and Child–Pugh class. Relevant perioperative data, the timing between TIPS and surgery and outcomes data was collected.

In addition, each study’s objectives were extracted as reported in the included studies, and this information was documented in the Additional file 1: Table S1.

### Risk of bias: critical appraisal

Different tools were used to appraise the studies and critically evaluate their risk of bias. For case series and comparative studies, we used the methodological index for non-randomized studies (MINORS) tool to assess their quality and internal validity [13]. MINORS critically appraises non-randomized studies across eight methodological domains in cases of observational studies without a comparison group (i.e., case series). For comparative studies, four additional items are evaluated. Each item in the MINORS tool was scored as 0: if not reported (Red: high risk of bias); 1: reported but inadequate (Yellow: unclear risk of bias); and 2: reported and adequate (Green: low risk of bias). The methodological quality of case reports was critically appraised through the Joanna Briggs Institute (JBI) critical appraisal tool for case reports. The results of the appraisal of research evidence are presented in detail in the Additional file 1: Figs. S1 and S2.

### Data synthesis: meta-analysis

The information collected from each study was summarized descriptively to chart the available literature. When available, we extracted data from comparative studies on the outcomes of interest: mortality and LREs. First, the number of individuals who did and did not experience the outcome in the treatment and control groups of each study was extracted into a 2 × 2 table. Then, a DerSimonian and Laird random-effects meta-analysis was performed to assess the overall outcomes of TIPS compared to non-TIPS groups. Heterogeneity was evaluated using the *I*^2^ test. An *I*^2^ > 75% revealed high heterogeneity. All analyses were performed in Stata statistical software.

## Results

**Fig. 1:**
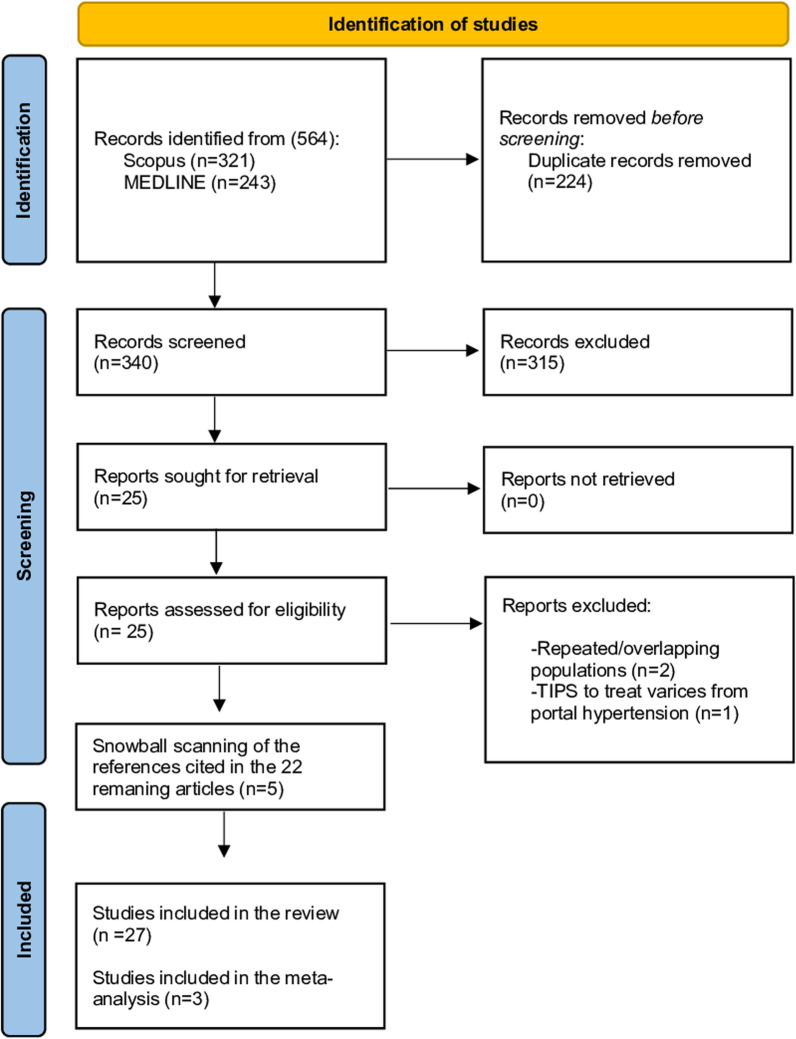
PRISMA flow diagram

Electronic database searching found 564 records (titles and abstracts), of which 25 were eligible for inclusion in this SR. In the full-text review, three studies were excluded, leaving 22 articles for inclusion. After conducting a snowball scanning of the references cited in these 22 articles, five additional references were found. Therefore, twenty-seven articles were finally included in the SR [14–40]. Of these, four were comparative studies, of which three were evaluated as appropriate to combine in meta-analyses [19, 37, 40]. Figure 1 shows the PRISMA diagram for the selection of the studies.

### Characteristics of the included studies

As shown in Table 1, the 27 articles included in this SR were published between 1995 and 2022. Of these, eleven recruited participants are from the USA. Fifteen were from Europe: Italy (*n* = 4), France (*n* = 4), UK (*n* = 4), Spain (*n* = 2), and Germany (*n* = 1); the remaining study was from Canada. More than half (*n* = 15, 55%) of the articles were case reports, eight (30%) were case series, and four (15%) were comparative studies.

**Table 1:** Study level data (study characteristics and patients’ information)

Author/y	Country	Study type	Cirrhosis etiology	Child-stage	Total pts/pts TIPS	Preoperative/postoperative	TIPS indication	n pts who attained surgery with preoperative TIPS	Age (TIPS group)	Condition requiring surgery	Type of surgery	Emergency-elective?	Timing between TIPS and surgery	Survival—mortality
Moulin 1995	France	CR	Alcohol	Child-B	1/1	1/0	Prophylactic	1	62	Adenocarcinoma of the esophagus	Endoscopic laser resection for adenocarcinoma of the esophagus: 1	Elective	120 days	Alive at 1-year follow-up
Amstrong 1998	USA	CR	Alcohol	Child-C	1/1	1/0	Prophylactic	1	55	Perforated diverticular disease	Sigmoid colectomy with a proximal diverting colostomy for diverticulitis: 1	Emergency	NR	Alive
Guglielmi 1999	Italy	CR	Alcohol	Child-B	1/1	1/0	Prophylactic	1	57	Gastric cancer	Endoscopic mucosal resection for gastric cancer: 1	Elective	30 days	Alive at 6-month follow-up
Azoulay 2001	France	CS	Alcohol: 5, HCV: 2	Child-A: 5, Child-B: 1, Child-C: 1	7/7	7/0	Prophylactic: 4, TIPS for ascites: 3	6	54 (54–67)	Colon cancer: 2, kidney cancer: 1, esophageal cancer: 1, aortic aneurysm: 1, heart cancer: 1, colostomy reversal: 1	Cancer resection: 5 (colon: 2, kidney: 1 esophageal: 1, heart: 1); aortic aneurysm repair: 1, colostomy reversal: 1	All elective	NR	One patient died
Grubel 2002	USA	CR	Alcohol and HCV	Child-C: 2	2/2	2/0	Prophylactic: 2	1	51 and 43	Colon cancer and kidney cancer	Sigmoid colectomy for colon cancer in 1; Nephrectomy for renal cancer in 1	Elective	21 and 56 days	The two patients remained alive at 1 year follow-up
Norton 2003	UK	CR	Alcohol	Child-A	1/1	1/0	Prophylactic	1	41	Gastric cancer	Gastrectomy for gastric cancer in 1	Elective	7 days	Alive at 6 months post-procedure
Fagan 2004	USA	CS	Alcohol: 2, HCV: 1	Child-B: 3	3/3	2/1	TIPS for ascites: 3 (one postop)	3	62, 65 and 51	Ruptured umbilical hernia	Umbilical herniorrhaphy for ruptured umbilical hernia in 3	All emergency	0 and 8 days. One pt had TIPS immediately after the surgical procedure	All alive in follow-up visits (3, 5 and 13 months)
Gil 2004	Spain	CS	HCV: 3	Child-A:2, Child-B:1	3/3	3/0	Prophylactic: 3	3	63, 70 and 60	Colon cancer: 1, gastric cancer: 1 and pancreas cancer: 1	Right hemicolectomy for colon cancer in 1, subtotal gastrectomy for gastric cancer in 1, pancreatoduodenectomy for pancreas cancer in 1	Elective	30, 45, and 14 days	Alive until hospital discharge
Catalano 2005	Italy	CR	Alcohol	Child-A	1/1	1/0	Prophylactic	1	63	Gastric adenocarcinoma	Subtotal distal gastrectomy for gastric cancer in 1	Elective	30 days	Alive at 12 months
Semiz-Oysu 2007	USA	CR	Alcohol: 1, NAFLD: 2	Child-A/B: 1/1	2/2	2/0	TIPS LRE: 2	2	68 and 61	Aortic stenosis and coronary artery disease in 2	Coronary artery bypass + aortic valve replacement in 1, coronary artery bypass in 1	Emergency: 1, elective:1	30 and 10 days	All alive at 6 months
Vinet 2006	Canada	Comparative study	Alcohol: 7	Median Child score: 7.7 (6–10)	35/18	18/0	Prophylactic: 13, TIPS for bleeding: 5	18	58 (14)	Colon cancer: 8, gastric antral vascular ectasia: 3, ulcerative colitis: 2, gastric cancer: 2, ampulloma: 1, small bowel stenosis: 1, kidney cancer: 1	Colectomy: 10, duodenopancreatectomy: 1, gastrectomy: 5, nephrectomy: 1, small bowel resection: 1	Elective	72 (21) days	1 year mortality: 8 (44%)
Kim 2009	USA	CS	Alcohol: 4, HCV: 4, PSC: 3, cyptogenic: 2, NASH: 2, portal vein thrombosis: 1, biliary atresia: 1	Child-A/B/C: 2/16/7	25/25	25/0	Prophylactic: 6, TIPS for ascites: 9, TIPS for bleeding: 10	25	49 (12)	Hernia: 7, gastro-intestinal perforation: 4, aortic stenosis: 2, colonic neoplasms: 2, kidney cancer: 1, cholangiocarcinoma: 1, testicular cancer: 1, bleeding cecal varices: 1, toxic megacolon: 1, mesenteric fibromatosis: 1, Ischemic Roux-en-Y: 1, arteriovenous malformation of the spleen: 1, pulmonary nodule: 1, pulmonary tuberculosis: 1	Gastrointestinal resections: 7, hernia repair: 6, exploratory laparotomy/lysis of adhesions: 4, aortic valve replacement: 2, splenectomy: 2, lung resection: 2, esophageal repair: 1, nephrectomy: 1	Emergency: 14, elective: 11	NR	1 year mortality: 7
Schlenker 2009	USA	CS	Alcohol: 3, PBC: 2, cryptogenic: 1, HCV: 1	Child A/B/C: 3/4/0	7/7	7/0	Prophylactic:7	7	56 (54–60)	Pelvic mass: 2, gastric/colon cancer: 2, complicated ovarian cyst: 1, cervical dysplasia: 1, renal cell carcinoma: 1	Salpingo-oophorectomy: 4, Gastrectomy/colectomy: 2, Nephrectomy: 1	Elective	13 (3–17) days	1 year mortality: 0
Minicozzi 2010	Italy	CR	Alcohol	Child-B	1/1	1/0	Prophylactic	1	70	Abdominal aortic aneurism and colon cancer	Endovascular aneurysm repair + laparoscopic right colectomy in 1	Elective	21 days	Alive at one year
Telem 2010	USA	CS	NR	NR	21/6	6/0	Prophylactic:6	6	NR	Ruptured/incarcerated hernias	Herniorrhaphy for ruptures incarcerated hernias in 1	Emergency	1 day	NR
Theruvath 2010	USA	CR	NR	Child-B	1/1	1/0	Prophylactic	1	55	Insulinoma/sigmoid adenocarcinoma	Enucleation of the pancreatic endocrine tumor and resection of sigmoid colon cancer in 1	Elective	2 days	Discharged home on day 10
Becq 2015	France	CR	NAFLD	NR	1/1	1/0	TIPS for ascites	1	67	Gastric antral vascular ectasia	Antrectomy for gastric antral vascular ectasia in 1	Elective	90 days	Alive at 3 months
Liverani 2015	Italy	CR	NR	Child-B	1/1	1/0	Prophylactic	1	80	Gastric cancer	Gastrectomy for gastric cancer in 1	Elective	30 days	Discharged home on day 14
de Andres 2016	Spain	CR	Alcohol	Child-A	1/1	1/0	Prophylactic	1	66	Achalasia	Laparoscopic Heller myotomy + dor fundoplication + cholecystectomy in 1	Elective	42 days	Alive at 6-months
Jabbar 2016	UK	CR	Congenital liver fibrosis	NR	1/1	1/0	Prophylactic	1	49	Common bile duct mass: bile duct adenoma with high frade dysplasia	Whipple procedure in 1	Elective	NR	Discharged home on day 28
Schmitz 2019	USA	CS	NASH: 9, alcohol: 6, HCV: 4, autoimmune hepatitis: 1, PBC	Child-A/B/C: 8/12/1	21/21	21/0	Prophylactic: 21	11	56.4 (8.8)	Hernia	"11 of the 21 patients who underwent TIPS creation had undergone the planned abdominal operation" Hernia repair in 11	Elective	NR	NR
Tabchouri 2019	France	Comparative study	Alcohol: 53, HCV: 6, NASH: 4, other: 3	Child-A/B/C: 40/24/2	134/66	66/0	Prophylactic: 66	56	60.9 (38–81)	NR	Colorectal surgery (30), Upper GI and pancreatic surgery (9), Hernia (8), Cholecystectomy (3), Other (6)	Elective	NR	30/90-day mortality: 1/4
Goel 2020	UK	CS	Alcohol: 8, NAFLD: 4, PSC: 3, other: 6	Child-A: 15, Child-B: 6	21/21	21/0	Prophylactic: 21	18	55 (33–76)	Colon cancer: 15, inflammatory bowel disease: 4, cystic fibrosis: 1, Alport's syndrome: 1	Colectomy (15), lung transplant (1), others (5)	Elective	38 (5–315) days	One year mortality = : 11 (52%)
Masood 2020	USA	CR	HCV	NR	1/1	1/0	Prophylactic	1	65	Colon cancer	Laparoscopic right hemicolectomy	Elective	60 days	Alive until hospital discharge
Aryan 2022	USA	Comparative study	NASH: 9, HCV: 6, alcohol: 7, NR: 2	NR	41/24	17/7	Prophylactic: 17, TIPS for LRE: 7 (7 postop)	24	52.8 (11.9)	Hernias: 22, cholecystitis: 1, spleen disease: 1	Hernia repair (21), bowel resection (5), cholecystectomy (1), other (1)	6 emergent hernia repairs	median 28 days (range: 4–202)	30 day mortality = 0%
Chang 2022	Germany	Comparative study	Alcohol: 32, viral: 4, other: 9	Child-A: 10, Child-B: 35	90/45	45/0	TIPS for LRE (variceal bleeding/ascites): 45	45	63 (43–80)	NR	Hernia repair (11), cholecystectomy (5),other hepatobiliary (5), bowel resection (2), orthopedic surgery (15), vascular surgery (2), other (5)	Emergency: 7, elective: 38	6 (0–101) months	8 patients died in the TIPS group
Kapeleris 2022	UK	CR	Alcohol: 1, autoimmune hepatitis with PSC: 1	Child-A: 1, Child-B: 1	2/2	2/0	Prophylactic: 2	1	56 and 36	Sigmoid adenocarcinoma and ulcerative colitis	Colon resection for sigmoid adenocarcinoma and ulcerative colitis in 1	Elective	NR	NR

*NR* not reported, *pts* patients, *CR* case report, *CS* case series, *HCV* hepatitis C virus, *NAFLD* non-alcoholic fatty liver disease, *PBC* primary biliary cirrhosis, *PSC* primary sclerosing cholangitis, *NASH* non-alcoholic steatohepatitis

Additional file 1: Table S1 presents each study’s objectives. This information reveals that the studies were homogeneous regarding the populations and the aims for which TIPS were created. In all studies, TIPS was placed as a preoperative adjunct to achieve portal decompression under the assumption that portal pressure reduction would diminish the risk of intraoperative and postoperative complications.

### Characteristics of participants

The studies in this SR recruited 426 patients. Of these patients, 264 underwent perioperative TIPS creation. The remaining patients were controls without TIPS, of which 15 were reported in a case series of patients with refractory ascites and hernia requiring surgery. However, no comparisons were made between groups in this case series [38]. Of note, in two studies reporting data from 27 patients with TIPS, eight subjects (n = 8) underwent postoperative TIPS creation, immediately after surgery. Therefore, 256 patients underwent preoperative TIPS to achieve portal decompression in anticipation of surgery.

As shown in Table 1, most patients were near or at the age to be classified as “senior adults” (60 years and above) and had cirrhosis of different etiologies, of which alcohol-related cirrhosis was the most frequent.

Additional file 1: Table S2 overviews portal hypertension features (including HVPG) and LREs. Overall, patients had clinical signs of clinically significant portal hypertension by either documented endoscopic proof of esophageal varices or previous episodes of variceal bleeding (see Additional file 1: Table S2 for detailed data). Moreover, 23 studies reported a history of a previous LRE, reflecting a high burden of decompensated cirrhosis in the included studies.

As mentioned above, 256 patients underwent preoperative TIPS, but not all TIPS insertions were performed pre-emptively to surgery. Of these patients, 70% (*n* = 179) underwent preoperative TIPS placement in a bridge to surgery. In contrast, 77 underwent preoperative TIPS placement as a therapeutic tool for an LRE that was present/in progress when surgery was schedule/planned, most commonly ascites and/or variceal bleeding (Table 1). Information regarding the type of stent used for TIPS creation was available in 12 studies. The use of a non-covered (Wallstent^®^) and an expanded polytetrafluoroethylene (ePTFE)-covered stent (Viatorr^®^) were reported in 6 and 3 studies, respectively. The remaining three studies reported using both types of stents (either Wallstent^®^ or Viatorr^®^ stent). The proportion of patients using one or another stent type was not available.

**Fig. 2:**
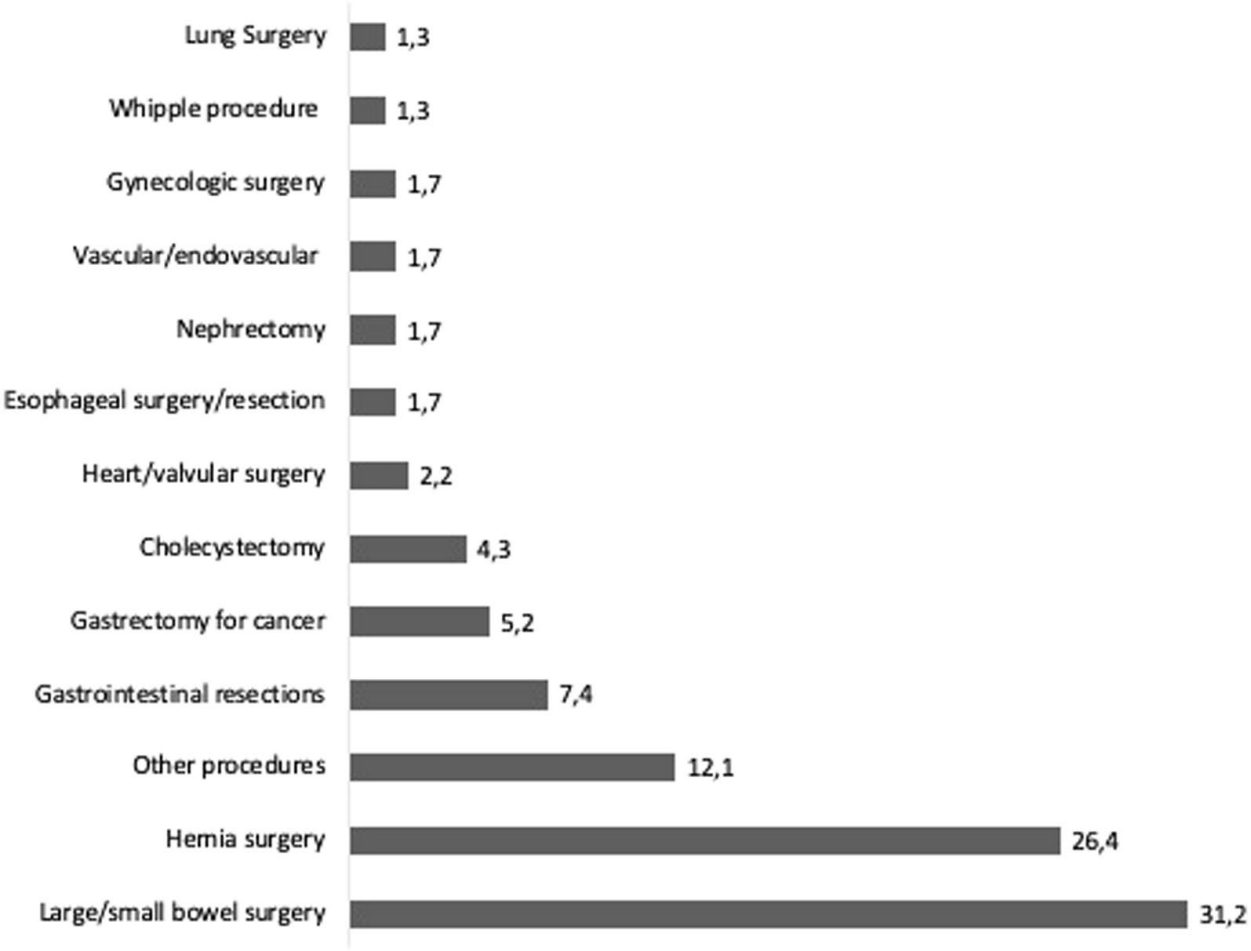
Types and percentages of surgical interventions performed

The conditions requiring surgery and the surgical interventions performed are detailed in Table 1. Also, as shown in Fig. 2, there were abdominal, thoracic, gynecologic, and vascular/endovascular procedures performed with preoperative TIPS. Tumor resection surgery was the most common, followed by non-oncologic gastrointestinal procedures, including hernia repair. In addition, there were cases of aortic aneurysm repair (*n* = 2), aortic valve replacement (*n* = 3), and coronary artery bypass (*n* = 2).

### Risk of bias

The results of the methodological quality assessment of the studies included in this SR are available in the Additional file 1: Figs. S1 and S2.

Overall, the case series and comparative studies were at risk of selection bias due to their retrospective nature and biased evaluation of endpoints. However, most of them presented a clearly stated aim and defined appropriate endpoints to the study’s aims. Regarding the comparative studies included in the MA, the intervention and comparison groups were contemporary, and it was likely that the groups had baseline equivalence.

The more common methodological pitfalls found across studies were the absence of prospectively collected data and the issues associated with study endpoints’ definition and evaluation. Also, in four studies, it was unclear whether the follow-up period was appropriate for the captured and reported outcomes (see Additional file 1: Fig. S1).

### Outcomes and meta-analysis

Surgery attainment/execution after TIPS was reported in 231 (90%) out of the 256 patients that underwent preoperative TIPS. From the 231 surgical procedures performed with preoperative TIPS, 38 (16%) were classified as emergency surgeries.

Four studies had control group data. However, one study [15] included seven patients that underwent perioperative TIPS in the postoperative period. This study was not considered for MA. In the remaining three comparative studies, patients who underwent preoperative TIPS were compared to controls with cirrhosis who underwent surgery without TIPS. Relevant clinical and outcome data from these articles are available in Table 2.

**Table 2:** Data from comparative studies included in the meta-analysis

	Chang 2022		Tabchouri 2019		Vinet 2006	
	TIPS (*n* = 45)	Control (*n* = 45)	TIPS (*n* = 66)	Control (*n* = 68)	TIPS (*n* = 18)	Control (*n* = 17)
Age	63 (43–80)	64 (40–77)	60.9 (38–81)	65.8 (42–80)	58 (14)	62 (12)
Sex (female), *n* (%)	13 (29%)	13 (29%)	16 (24%)	14 (20%)	4 (22%)	6 (35%)
Cirrhosis etiology	Alcohol: 32 (71%), Viral hep: 4 (9%), other: 9 (20%)	Alcohol: 32 (71%), Viral hep (9%): 4, other: 9 (20%)	Alcohol: 53 (80.3%), Hep C: 6 (6.8%), NASH: 4 (6%), other: 3 (3.4%)	Alcohol: 50 (73.5%), Hep C: 7 (10.2%), NASH: 3 (4.4%), other: 8 (11.8%)	Alcohol: 7 (39%)	6 (35%)
MELD score	11 (6–17)	10 (6–18)	11 (6–21)	11 (6–25)	NR	NR
Child–Pugh class	A: 10, B: 35	A: 10, B: 35	A: 40 (60%), B: 24 (37%), C: 2 (3%)	A: 47 (69%), B: 21 (31%), C: 0	Pugh score: 7.7 (6–10)	Pugh score: 6.2 (5–9)
Surgical procedures	Non-visceral: 21 (47%), Visceral: 24 (5,%)	Non-visceral: 21 (47%), Visceral: 24 (5,%)	Colorectal: 24 (51.5%), upper GI and pancreatic: 12 (18.2%), Hernia and incisional hernia: 9 (13.6%), cholecystectomy: 5 (7.6%), other: 6 (9.1%)	Colorectal: 38 (57,4%), upper GI and pancreatic: 4 (5,9%), Hernia and incisional hernia: 9 (13.2%), cholecystectomy: 10 (14,7%), other: 7 (10,2%)	Colectomy: 10, duodenopancreatectomy: 1, gastrectomy: 5, nephrectomy: 1. small bowel resection: 1	Colectomy: 13, duodenopancreatectomy: 2, gastrectomy: 1, nephrectomy: 1, small bowel resection: 0
Emergency surgery, n (%)	7 (16%)	7 (16%)	NR	NR	NR	NR
Control group selection method	1:1 Propensity score matched groups		Inverse probability weighting—propensity score		Not matching. Retrospective cohort study including all patients who underwent TIPS placement before an elective abdominal operation and compared to all cirrhotic patients who underwent elective abdominal surgeries without TIPS between 1992 and 2002	
Postop Outcomes	Data for matched groups (TIPS: 45 vs Controls: 45)		Data for matched groups (TIPS: 56 vs controls: 68)		Data for all the cohort (TIPS: 18 vs. Controls: 17)	
Ascites	15 (33%)	25 (56%)	11 (20.4%)	26 (38.2%)	NR	NR
HE	5 (11%)	4 (9%)	NR	NR	4 (22%)	5 (29%)
Perioperative blood transfusions	11 (24%)	20 (44%)	17 (30.4%)	9 (13.2%)	6 (33%)	7 (41%)
ACLF	4 (8.9%)	13 (28.9%)	8 (14.5%)	2 (2.9%)	3 (16%)	3 (18%)
90-day mortality	5 (11.1%)	11 (24%)	4 (7.5%)	5 (7.8%)	6 (33%)	4 (23%)

**Fig. 3:**
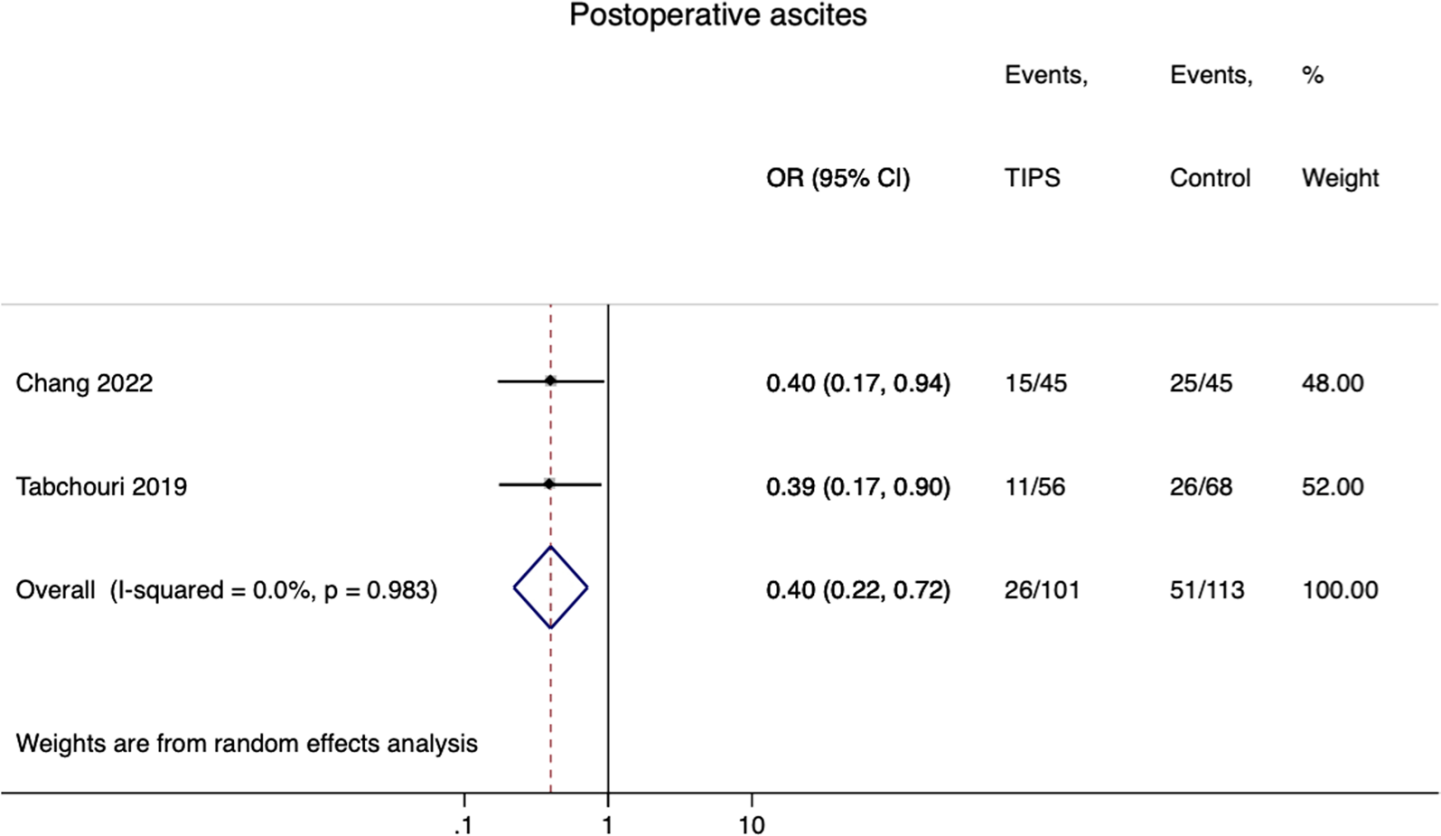
Forest plot for ascites (TIPS vs. non-TIPS)

Random effects MA showed significantly lower odds of postoperative ascites if preoperative TIPS was created (2 studies: OR = 0.40, 95% CI 0.22–0.72; I2 = 0%) (Fig. 3). We acknowledge that the data combined for the MA of ascites came from studies that used propensity score matching techniques as the method for selecting controls.

**Fig. 4:**
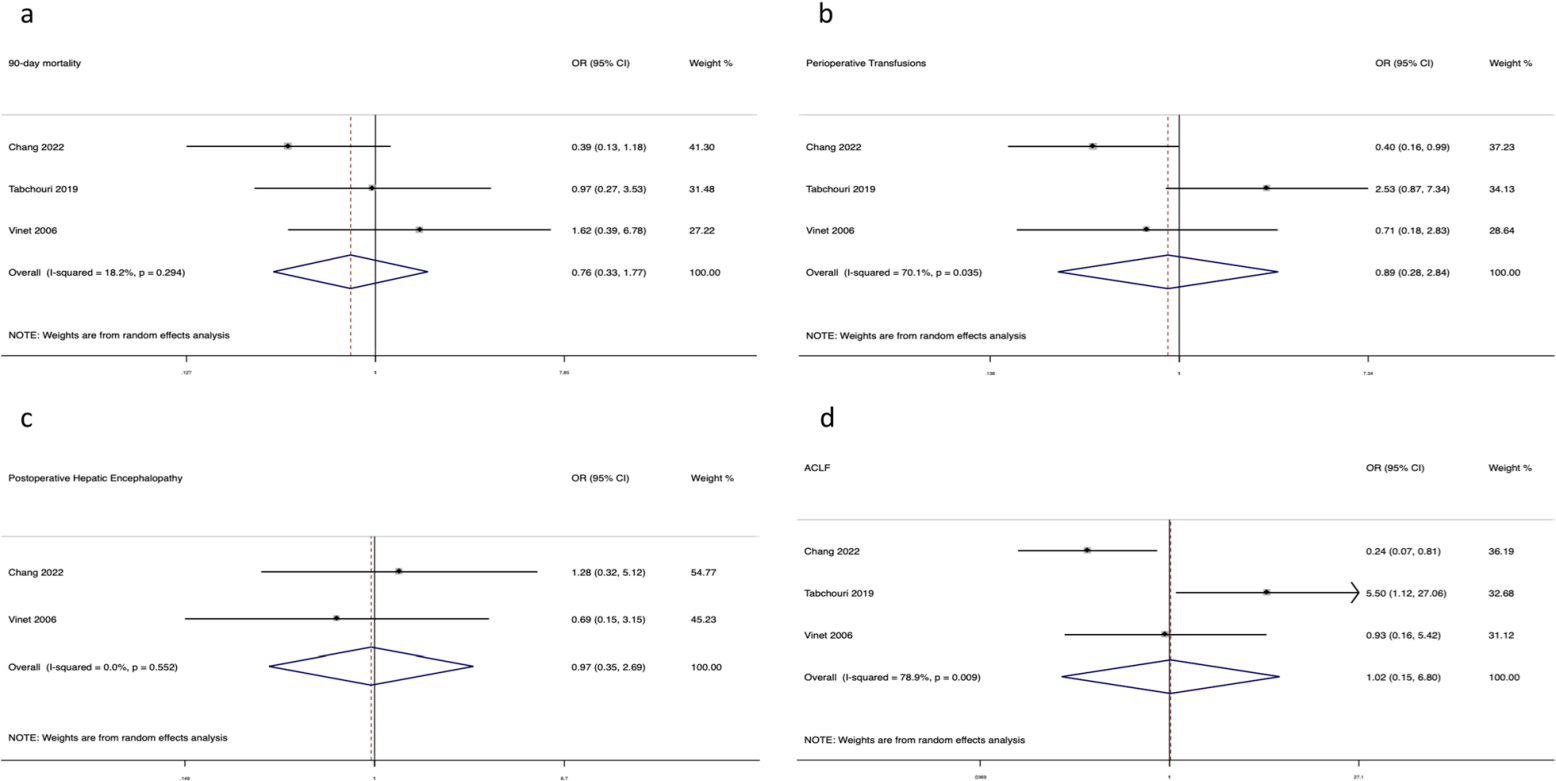
Forest plots for the outcomes of interest: **a** 90-day mortality; **b** Perioperative transfusions; **c** Postoperative hepatic encephalopathy; **d** Postoperative ACLF

In contrast, random effects MA (Fig. 4) found no significant differences in 90-day mortality (3 studies: OR = 0.76, 95% CI 0.33–1.77; I2 = 18.2%) (Fig. 4a), perioperative transfusion requirement (3 studies: OR = 0.89, 95% CI 0.28–2.84; I2 = 70.1%) (Fig. 4b), postoperative hepatic encephalopathy (2 studies: OR = 0.97, 95% CI 0.35–2.69; I2 = 0%) (Fig. 4c) and postoperative ACLF (3 studies: OR = 1.02, 95% CI 0.15–6.8, I2 = 78.9%) (Fig. 4d).

## Discussion

This meta-analytic review assessed the outcomes of cirrhotic patients that underwent surgery with preoperative TIPS as an adjunct to decompress the portal system. Only studies reporting observational data were found. Three key points can be extracted from our work: first, it is feasible to deploy TIPS as a bridge to surgery as it appears not to jeopardize the attainment/execution of surgical procedures. The data shows that 90% of patients who underwent preoperative TIPS attained/achieved surgery. Second, different types of operations were performed with preoperative TIPS and no differences in LREs were found. Therefore, and acknowledging that available data are limited, it appears that the type of procedure should not be considered a contraindication to offer surgery to cirrhotic patients with PH, even in cases where emergency surgery is needed. And third, the preoperative decompression of the portal circulation through TIPS may have a beneficial effect on the occurrence of postoperative ascites. However, we found no significant effect of preoperative TIPS on other outcomes such as perioperative transfusions, and postsurgical liver events other than ascites, i.e., hepatic encephalopathy, ACLF, and mortality.

The data assembled show that TIPS can be successfully deployed or performed in a variety of surgical procedures and settings across multiple surgical specialties/disciplines. As shown in Fig. 2, abdominal, thoracic, gynecologic, and vascular/endovascular procedures were all performed with preoperative TIPS. Tumor resection surgery was the most common procedure, followed by non-oncologic gastrointestinal procedures such as hernia repair. In addition, there were cases of aortic aneurysm repair, aortic valve replacement, and coronary artery bypass in which TIPS was utilized. While further research is needed to fully understand the effect of TIPS on surgical and postoperative outcomes, surgeons should consider the use of TIPS as a potential tool in the surgical management of cirrhotic patients, including emergency and surgical rescue situations [41]. As such, it should be included in the armamentarium of surgeons practicing in these settings and should also be included in the research agenda of general and emergency surgeons worldwide. It is important to note, however, that the use of TIPS should be restricted to centers with a high volume of cases and a demonstrated expertise in the preoperative deployment of TIPS. Effective communication between the surgeon, interventional radiologist, and hepatologist is also essential for the achievement of optimal outcomes.

We found that preoperative TIPS resulted in lower odds of postoperative ascites after combining data derived from propensity score matching techniques in a random-effects meta-analysis. This is not surprising as TIPS directly acts on the main driver of ascites occurrence by decompressing the hepatic sinusoids and reducing portal pressure. Although no randomized studies have been performed for TIPS in perioperative medicine, preoperative TIPS may protect an already exhausted liver vascular structure unprepared to tolerate the pathophysiological changes that occur as a stress response to surgery [5]. The effects of TIPS could be of greater significance in patients requiring longer or more invasive surgeries as these procedures elicit greater stress responses. However, further prospective (and particularly randomized) studies are required to validate our findings regarding postoperative outcomes in patients undergoing preoperative TIPS. These studies should also evaluate if preoperative TIPS could reduce the hospital cost derived from postoperative ascites care and other liver-related events.

In contrast to the role that preoperative TIPS may have in reducing the odds of postoperative ascites, our analyses found no differences in mortality, perioperative transfusions, hepatic encephalopathy, and ACLF. Since the studies included in our MA are retrospective, there would be an unavoidable selection bias, where both TIPS candidates and those cirrhotic patients that did not underwent to a TIPS creation had a preserved liver function that would impact directly in the risk of developing an LRE during and after the surgery.

In our opinion, future studies should be focused on three priorities: 1. To assess the effectiveness of preoperative TIPS in randomized clinical trials, as mentioned above, and specifically if there are reduced costs resulting from less postoperative ascites, 2. To assess the factors associated with not achieving/attaining surgery after preoperative TIPS and, 3. To identify predictors of postoperative liver decompensation in patients who underwent surgery with preoperative TIPS. Specifically, is there a role for determining hepatic venous pressure gradient in the perioperative care of cirrhotic patients undergoing major surgery, and the predictive performance of noninvasive tests for postoperative outcomes. Moreover, given the well-known advantages of PTFE-covered stent grafts in terms of hepatic encephalopathy and survival [42], future research should also assess whether the prophylactic use of these endoprostheses positively impacts the outcomes of patients undergoing elective or emergency surgery. These data may inform decision-making and clinical guidelines development. Meanwhile, preoperative TIPS should be employed cautiously in well-selected patients and performed by teams with experience in the procedure and demonstrated high-case volume.

### Limitations

This report has limitations, and the results should be interpreted in the context of the study design. First, the meta-analysis was based on observational data, thereby making it prone to meta-bias [43] and limiting the applicability of its results. Second, certain relevant data were not systematically reported in most studies and thus, could not be analyzed in our SR + MA, i.e., Child–Pugh and MELD scores of patients before undergoing TIPS, underlying cause of cirrhosis, hepatic venous pressure gradient, or specifics on LRE after surgery. To overcome the heterogeneity in outcome reporting, future surgical research studies evaluating the effect of preoperative TIPS in patients with advanced liver disease should include a core outcome set [44] to help guide the appropriate standardization and reporting of outcomes relevant to health professionals, patients, and health care efficiency.

Third, although a number of articles were reviewed encompassing different study designs, outcomes, and settings, there was a notable lack of solid comparative effectiveness-oriented studies, including randomized controlled clinical trials, of which we could not find any. This might be because preoperative TIPS is still considered an unusual procedure as, unfortunately, no randomized trials have been performed in this setting, thus, diminishing the odds of implementing it into clinical practice.

Despite its limitations, this study synthesized data from the available literature to assemble a range of examples of what happened when TIPS was implemented and used as a preemptive preoperative adjunct in different surgical scenarios. Therefore, the results presented herein should serve as the starting point for more detailed investigations focusing on assessing the effectiveness of preoperative TIPS from randomized studies. Hence, this report should not be used to implement changes in clinical practices. Instead, these results should inform research endeavors on the same matter. Endeavors in which emergency general surgeons [45] could play a fundamental role in advancing the field of endovascular emergency procedures worldwide [46].

## Conclusion

Preoperative TIPS appears to be safe in cirrhotic patients with PH who undergo elective and emergency surgery and may have a role in postoperative ascites control. Since available evidence to date is insufficient to provide any recommendation of the TIPS role in this setting, our results underscore the unmet need for prospective randomized studies to elucidate the effect of preoperative TIPS on liver outcomes and mortality after surgery in cirrhotic patients.

## Supplementary Information

**Additional file 1.** Supplementary File.

## Data Availability

The datasets and Stata commands used during the current study can become available from the corresponding author on reasonable request.

## Abbreviations

SR: Systematic review
MA: Meta-analysis
PH: Portal hypertension
HVPG: Hepatic venous pressure gradient (HVPG),
TIPS: Transjugular intrahepatic portosystemic shunt
LRE: Liver-related events
ACLF: Acute on chronic liver failure
MINORS: Methodological index for non-randomized studies
