# Supplementary material for: Unlocking the potential of TIPS placement as a bridge to elective and emergency surgery in cirrhotic patients: a meta-analysis and future directions for endovascular resuscitation in acute care surgery
Source: World J Emerg Surg. 2023 Apr 17;18:30. doi: 10.1186/s13017-023-00498-4 (PMC10111768; doi:10.1186/s13017-023-00498-4)
Supplement: Supplementary file 1 — Additional file 1. Supplementary File. [file 13017_2023_498_MOESM1_ESM.docx]

# Supplementary File

The present electronic supplementary material was prepared for the manuscript **“Unlocking the Potential of TIPS Placement as a Bridge to Elective and Emergency Surgery in Cirrhotic Patients: A Meta-Analysis and Future Directions for Endovascular Resuscitation in Acute Care Surgery”**. For questions about the information herein contained, please get in touch with the corresponding author:

Ramiro Manzano-Nunez MD

e-mail address: [ramiro.manzano@autonoma.cat](mailto:juanmanuel.pericas@vallhebron.cat)

Universitat Autònoma de Barcelona

Barcelona, Spain

This supplement contains the following items:

# Search Strategies

1. **Risk of Bias within studies:**

**Figure S1.** Risk of bias assessment of case series and comparative studies according to MINORS

**Figure S2.** Critical appraisal of case reports according to the Joanna Briggs Institute Critical appraisal tool for case reports

# Table S1 Study’s objectives/case characteristics as reported in each manuscript

1. **Table S2 – Patients’ features demonstrating portal hypertension and history of liver- related events (LREs)**

# Search strategies:

- 1. Scopus (results= 321 documents):

( TITLE-ABS-KEY ( {transjugular intrahepatic portosystemic shunt} ) OR TITLE-ABS-

KEY ( tipss ) OR TITLE-ABS-KEY ( {percutaneous intrahepatic portosystemic

shunt} ) AND TITLE-ABS-KEY ( {extrahepatic surgery} ) OR TITLE-ABS-KEY ( {major surgery} ) OR TITLE-ABS-KEY ( "preoperative" ) OR TITLE-ABS-

KEY ( "perioperative" ) OR TITLE-ABS-KEY ( {abdominal surgery} ) )

- 1. MEDLINE (results=243 documents):
     1. Exp Portasystemic Shunt, Transjugular Intrahepatic/
     2. Percutaneousous transhepatic intrahepatic portosystemic shunt.mp.
     3. Percutaneous portosystemic shunt*.mp.
     4. Transjugular intrahepatic Portosystemic Shunt*.mp.
     5. Percutaneous Intrahepatic Portosystemic Shunt*.mp.
     6. Intrahepatic Portosystemic Shunt*.mp.
     7. TIPSS.mp.
     8. Prophylactic TIPS.mp.
     9. Preoperative transjugular intrahepatic portosystemic shunt*.mp.
     10. Perioperative transjugular intrahepatic portosystemic shunt*.mp.
     11. Pre-surgical.mp.
     12. Abdominal surgery.mp.
     13. Exp General surgery/
     14. Major surgery.mp.
     15. Extrahepatic surgery.mp.
     16. Exp Perioperative Care/
     17. Exp Perioperative Period
     18. Perioperative.mp.
     19. 1 or 2 or 3 or 4 or 5 or 6 or 7 or 8 or 9 or 10 20. 11 or 12 or 13 or 14 or 15 or 16 or 17 or 18

21. 19 and 20

# Risk of Bias Within studies:

**Figure S1. Risk of bias assessment of case series and comparative studies according to MINORS**

|  |  |  |  |  |  |  |  |  | **In the case of comparative studies** | | | | |
| --- | --- | --- | --- | --- | --- | --- | --- | --- | --- | --- | --- | --- | --- |
|  | **Comparative study?** | **A clearly stated aim** | **Inclusion of consecutive patients** | **Prospective collection of data** | **Endpoint appropriate to the study aim** | **Unbiased evaluation of endpoints** | **Follow-up appropriate to the major endpoint** | **Loss to follow-up not exceeding 5%** | **A control group having the gold standard intervention** | **Contemporary groups** | **Baseline equivalence of groups** | **Prospective calculation of the sample size** | **Statistical analyses adapted to the study design** |
| **Azoulay 2001** | No |  |  |  |  |  |  |  |  |  |  |  |  |
| **Fagan 2004** | No |  |  |  |  |  |  |  |  |  |  |  |  |
| **Gil 2004** | No |  |  |  |  |  |  |  |  |  |  |  |  |
| **Vinet 2006** | Yes |  |  |  |  |  |  |  |  |  |  |  |  |
| **Kim 2009** | No |  |  |  |  |  |  |  |  |  |  |  |  |
| **Schlenker 2009** | No |  |  |  |  |  |  |  |  |  |  |  |  |
| **Telem 2010** | No |  |  |  |  |  |  |  |  |  |  |  |  |
| **Schmitz 2019** | No |  |  |  |  |  |  |  |  |  |  |  |  |
| **Tabchouri 2019** | Yes |  |  |  |  |  |  |  |  |  |  |  |  |
| **Goel 2020** | No |  |  |  |  |  |  |  |  |  |  |  |  |
| **Aryan 2022** | Yes |  |  |  |  |  |  |  |  |  |  |  |  |
| **Chang 2022** | Yes |  |  |  |  |  |  |  |  |  |  |  |  |

# Figure S2. Critical appraisal of case reports according to the Joanna Briggs Institute’s Critical appraisal tool for case reports

The JBI’s critical appraisal tools can be found on the JBI’s website ([https://jbi.global/critical-appraisal-](https://jbi.global/critical-appraisal-tools) [tools](https://jbi.global/critical-appraisal-tools)). The critical appraisal tool for case reports assesses the quality and relevance of case reports by evaluating six characteristics that the reporting of case reports should include (see figure). Each domain is scored as Yes (green), No (red), and Unclear (yellow). The results of the latter evaluation are represented in the following figure:

|  | **Were patient’s demographic characteristics clearly described?** | **Was the patient’s history clearly described and presented as a timeline?** | **Was the current clinical condition of the patient on presentation clearly described?** | **Were diagnostic tests or assessment methods and the results clearly described?** | **Was the intervention(s) or treatment procedure(s) clearly described?** | **Was the post-intervention clinical condition clearly described?** | **Were adverse events (harms) or unanticipated events identified and described?** | **Does the case report provide takeaway lessons?** |
| --- | --- | --- | --- | --- | --- | --- | --- | --- |
| **Moulin 1995** |  |  |  |  |  |  |  |  |
| **Amstrong 1998** |  |  |  |  |  |  |  |  |
| **Guglielmi 1999** |  |  |  |  |  |  |  |  |
| **Grubel 2002** |  |  |  |  |  |  |  |  |
| **Norton 2003** |  |  |  |  |  |  |  |  |
| **Catalano 2005** |  |  |  |  |  |  |  |  |
| **Semiz-Oysu 2007** |  |  |  |  |  |  |  |  |
| **Minicozzi 2010** |  |  |  |  |  |  |  |  |
| **Theruvath 2010** |  |  |  |  |  |  |  |  |
| **Becq 2015** |  |  |  |  |  |  |  |  |
| **Liverani 2015** |  |  |  |  |  |  |  |  |
| **de Andres 2016** |  |  |  |  |  |  |  |  |
| **Jabbar 2016** |  |  |  |  |  |  |  |  |
| **Masood 2020** |  |  |  |  |  |  |  |  |
| **Kapeleris 2022** |  |  |  |  |  |  |  |  |

1. **Table S1 Study’s objectives or case characteristics as reported in each manuscript**

| **Author/y** | **Year** | **Objective/case characteristics** |
| --- | --- | --- |
| **Moulin 1995** | 1995 | "In the present report, we describe a patient in whom TIPS allowed palliative  laser resection of an adenocarcinoma of the esophagus covering esophageal varices" |
| **Amstrong 1998** | 1998 | "We report a case of perforated diverticular diseases of the sigmoid colon, with intra-abdominal abscess, in a patient with severe portal hypertension and varices. Three-dimensional reconstruction of theabdominopelvic computed tomography angiographic scan illustrated the need for preoperative portal system decompression with TIPS before sigmoid  diverticular disease resection |
| **Guglielmi 1999** | 1999 | "We report herein a cirrhotic patient with early gastric cancer, presenting with gastroesophageal varices and severe hypertensive gastropathy, who underwent TIPS to reduce portal hypertension and was subsequently treated  with endoscopy mucosal resection" |
| **Azoulay 2001** | 2001 | "The present study reports our experience with seven consecutive cirrhotic  patients deemed inoperable because of complicated severe portal hypertension who were prepared for major abdominal surgery by TIPS" |
| **Grubel 2002** | 2002 | "We observed two patients with Child-Pugh class C cirrhosis and severe portal  hypertension; one had been diagnosed with sigmoid colon cancer requiring |

|  |  | sigmoidectomy, the other, with renal cell carcinoma requiring nephrectomy. To limit their operative risk of bleeding from abdominal and retroperitoneal varices, transjugular intrahepatic portosystemic shunts (TIPS) were placed preoperatively to decompress the portal system and reduce blood flow  through portosystemic collaterals." |
| --- | --- | --- |
| **Norton 2003** | 2003 | "We describe a case in which curative gastric resection was performed for  carcinoma of the stomach after a preoperative TIPS and embolization of a large gastric varix in a patient with portal hypertension" |
| **Fagan 2004** | 2004 | "Here, we describe the combined use of TIPS and urgent umbilical hernia repair in 3 patients with refractory ascites. Two patients who presented with frank rupture of an umbilical hernia and leaking ascites were managed initially by TIPS followed by hernia repair. A third patient presenting with ascites and gangrenous intact skin overlying an umbilical hernia (indicating impending rupture) required emergent umbilical hernia repair followed by a TIPS  procedure postoperatively." |
| **Gil 2004** | 2004 | "The present study reports our experience with three cirrhotic patients with abdominal tumours deemed inoperable because of their portal hypertension  who were prepared for major abdominal surgery by TIPS." |
| **Catalano 2005** | 2005 | "We report the case of a 63-year-old male patient referred to our institution for recalcitrant epigastric pain in the setting of CTP A6 alcoholic liver cirrhosis. Endoscopy revealed the presence of a 3-cm ulcer located in the gastric antrum in association with Grade 2 distal esophageal varices with no red cherry spot marks. Histology of the bioptic sample was consistent with gastric adenocarcinoma. The patient was referred to preoperative TIPSS to reduce the surgical morbidity and allow for safe nodal"  dissection |
| **Semiz-Oysu 2007** | 2006 | "We performed TIPS procedure in two patients prior to cardiac surgery with the use of cardiopulmonary bypass in order to reduce the increased risks  associated with portal hypertension." |
| **Vinet 2006** | 2006 | "In the present study, using retrospective design, we evaluated the postoperative morbidity and mortality rates after major abdominal surgery in  cirrhotic patients with or without preoperative TIPS placement" |
| **Kim 2009** | 2009 | The aim of this study is to assess the outcomes in consecutive cirrhotic patients who had achieved portal decompression via a TIPS before major  abdominal or thoracic surgery |
| **Schlenker 2009** | 2009 | "Placement of TIPS also has been reported for a small number of patients with cirrhosis and portal hypertension undergoing abdominal surgery to prevent complications. We have had success with the use of TIPS for this indication  and report our experience" |
| **Minicozzi 2010** | 2010 | "This case shows that the use of minimally invasive procedures is effective and can improve the results of complex operations even in patients with severe cirrhosis". 70-year-old male affected by alcoholic cirrhosis underwent  neoadjuvant transjugular intrahepatic portosystemic shunt before surgery |
| **Telem 2010** | 2010 | "This study represents our institutional experience with the management and outcome after umbilical herniorrhaphy in one of the larger series of patients with advanced cirrhosis and refractory ascites. The purpose of our hospital- based study was to assess variables influencing short and long-term outcome  after herniorrhaphy" |
| **Theruvath 2010** | 2010 | "This report presents a case with neoadjuvant TIPS placement in a patient with Child-Turcotte-Pugh Class B cirrhosis and portal hypertension closely followed by surgical resection of an insulinoma and synchronous sigmoid  adenocarcinoma" |
| **Becq 2015** | 2015 | "We report the case of a 67-year-old cirrhotic patient who presented with GAVE related GIB, unresponsive to multiple endoscopic treatments. The patient had a good liver function (model for end-stage disease 10). After a  multidisciplinary meeting, a transjugular intrahepatic portosystemic shunt |

|  |  | (TIPS) procedure was performed, in order to treat the cirrhosis associated ascites. The outcome was successful. An antrectomy was then performed, with no recurrence of (gastrointestinal bleeding) GIB and no transfusion need during three months of follow up. In this case, the TIPS procedure achieved a complete ascites regression, allowing a safer surgical treatment of the GAVE-  related GIB." |
| --- | --- | --- |
| **Liverani 2015** | 2015 | "We retained preoperative the trans-jugular porto-systemic shunt in the patients with elevated portal pressure and gastric cancer to perform a gastrectomy more safely and to decrease morbidity and mortality of these  cases." |
| **de Andres 2016** | 2016 | "We present the case of a patient with achalasia and gastroesophageal varices due to alcoholic cirrhosis in whom a TIPS was inserted preoperatively and varices were embolized, with subsequent laparoscopic fundoplication and  Heller myotomy." |
| **Jabbar 2016** | 2016 | We report on a patient undergoing portal decompression via TIPS prior to a  whipple resection |
| **Schmitz 2019** | 2019 | "The goal of this study was to determine the percentage of patients who underwent abdominal operation following preoperative TIPS creation and to understand the relationship between preoperative TIPS and perioperative  outcomes" |
| **Tabchouri 2019** | 2019 | "The aim of this study was to compare the morbidity and mortality following elective extrahepatic abdominal surgery between cirrhotic patients with preoperative TIPS placement (in a bridge to surgery setting) and cirrhotic  patients without TIPS" |
| **Goel 2020** | 2020 | "We describe our experience of TIPS to facilitate nonhepatic surgery in the  setting of patients with liver cirrhosis and established portal hypertension" |
| **Masood 2020** | 2020 | "We report a caso of a patient with early-stage colon cancer and large caput medusae, who underwent Transjugular Intra-hepatic Postosystemic Shunt (TIPS) in order to decompress the periumbilical veins to allow for surgical  resection of the colon cancer" |
| **Aryan 2022** | 2022 | "We investigated the utility of perioperative TIPS in patients with cirrhosis undergoing abdominal surgery by analyzing postoperative complications  when compared to cirrhotics undergoing abdominal surgery without TIPS" |
| **Chang 2022** | 2022 | "This retrospective study aimed to investigate the impact of preoperative TIPS placement on ACLF development and mortality in patients with liver cirrhosis  undergoing surgery" |
| **Kapeleris**  **2022** | 2022 | "We present two patients who underwent TIPSS as a bridge procedure and  their outcomes" |

1. **Table S2 – Patients’ features demonstrating portal hypertension and history of liver-related events (LREs)**

| **Author/y** | **Yea r** | **Study type** | **HVPG**  **pre-TIPS (mmHg)** | **HVPG**  **post-TIPS (mmHg)** | **Presence/histo ry of esophageal varices before**  **TIPS?** | **History of previous LRE/decompensat ed cirrhosis?** |
| --- | --- | --- | --- | --- | --- | --- |
| **Moulin 1995** | 199  5 | CR | 25 | 8 | Yes: Grade 3 | Yes, variceal  bleeding |
| **Amstrong 1998** | 199  8 | CR | NR | NR | Yes: “large portal venous varices” | Yes, Child-C cirrhosis |
| **Guglielmi 1999** | 199  9 | CR | 32 | 22 | Yes, not  graded. | Yes, ascites |

| **Azoulay 2001** | 200  1 | CS | 18 (5)* | 9 (5)* | Yes, in all. Grade 2 in 3/7, grade 3 in 3/7. Not graded in 1 | Yes, history of variceal bleeding in 5/7 patients and a history of ascites in  3. |
| --- | --- | --- | --- | --- | --- | --- |
| **Grubel 2002** | 200  2 | CR | Case 1:  17  Case 2:  26 | Case 1: 8  Case 2: 14 | Case 1: history of variceal bleeding,  Case 2: history of variceal  bleeding | Yes. Case 1: ascites and variceal bleeding. Case 2: ascites, variceal bleeding, and HE. |
| **Norton 2003** | 200  3 | CR | 16 | 12 | Yes, presented with bleeding esophageal  varices | Yes, variceal bleeding. |
| **Fagan 2004** | 200  4 | CS | NR | NR | NR | Yes, ascites in all 3  patients |
| **Gil 2004** | 200  4 | CS | Case 1:  22  Case 2:  20,  Case 3:  28 | Case 1: 7  Case 2: 7,  Case 3: 7 | Yes: grade 3 in two cases.  Grade 2 in one case. | Yes, variceal bleeding in 1 patient. |
| **Catalano 2005** | 200  5 | CR | 20 | 5 | Yes: grade 2 | NR |
| **Semiz-Oysu 2007** | 200  6 | CR | Case 1:  19,  Case 2:  20 | Case 1: 5,  Case 2: 10 | Case 1: grade 3,  Case 2: grade 2 | Yes, ascites in 1 patient. |
| **Vinet 2006** | 200  6 | Comparativ e study | 21.4  (3.9) | 8.4 (3.4) | Yes, history of variceal bleeding in 5  patients. | Yes. History of ascites in 7/18. Previous HE=3/18. |
| **Kim 2009** | 200  9 | CS | 19.6  (5.5) | 8.7 (2.9) | NR | Yes, previous HE in 42%, history of  ascites in 71% |
| **Schlenker 2009** | 200  9 | CS | Case 1:  12,  Case 2: unknow n,  Case 3:  12,  Case 4:  9,  Case 5:  22,  Case 6: unknow n,  Case 7:  21 | Case 1: 4,  Case 2: unknown, Case 3: 8,  Case 4: 7,  Case 5:  10,  Case 6: 3,  Case 7: 8 | Yes, present in 3/7 patients. History of variceal bleeding in 2/7 patients. | Yes, previous LRE in 6/7 patients. |
| **Minicozzi 2010** | 201  0 | CR | 31 | 19 | Yes, not graded | NR |
| **Telem 2010** | 201  0 | CS | NR | NR | NR | Yes, previous LRE  in 4/6 patients. |

| **Theruvath**  **2010** | 201  0 | CR | 27 | 10 | Yes, not graded | Yes, ascites |
| --- | --- | --- | --- | --- | --- | --- |
| **Becq 2015** | 201  5 | CR | NR | NR | NR | Yes, ascites and  variceal bleeding |
| **Liverani 2015** | 201  5 | CR | NR | NR | Yes, history of variceal  bleeding | Yes, variceal bleeding |
| **de Andres 2016** | 201  6 | CR | NR | NR | Yes, not  graded. | Yes, ascites |
| **Jabbar 2016** | 201  6 | CR | NR | NR | Grade B esophageal  varices | NR |
| **Schmitz 2019** | 201  9 | CS | 14.3 (4.6)* | 4.9 (1.7)* | Yes, present in 18/21 (85.7%) | Yes, History of variceal bleeding: 5  (23%), History of  ascites: 13 (61%), History of HE: 7  (33%) |
| **Tabchouri 2019** | 201  9 | Comparativ e study | 13.8 (6-  23)** | 5.4 (1-  11)** | Yes: Grade 1 in 8 (14%), Grade  2 in 19 (33%),  Grade 3 in 21  (37%) | Yes, ascites in 20 (30%). |
| **Goel 2020** | 202  0 | CS | NR | Post TIPS gradient:  5 (1-13)** | NR | Yes, ascites or variceal bleeding in  4/21. |
| **Masood 2020** | 202  0 | CR | 11  mmHg | NR | Yes | NR |
| **Aryan 2022** | 202  2 | Comparativ e study | 16.3 (4.6)* | 5.3 (2.3)* | Yes, previous variceal bleeding in 8  (29%) | Yes. Ascites in 26 (93%), variceal  bleeding in 8  (29%), HE in 13  (46%) |
| **Chang 2022** | 202  2 | Comparativ e study | NR | NR | Grade 1: 11  (24%), Grade 2:  13 (29%),  Grade 3: 11  (24%) | Yes. History of ascites: 35 (77%). History of variceal bleeding: 18 (40%). History of HE: 8  (17%) |
| **Kapeleris 2022** | 202  2 | CR | Case 1:  33,  Case 2:  22 | Case 1: 7,  Case 2: 19 | Yes | Yes, ascites in 1 patient. |

**CS: Case series, CR: Case report, HE: hepatic encephalopathy, *mean and SD, **median and range, NR: not reported**
